# Free Midwives

**Published:** 1919-08-30

**Authors:** 


### FREE MIDWIVES.

### The Practice in Big Towns.

BEFORE deciding on a policy in regard to a midwifery service, the Hull Corporation asked the Town Clerk to make inquiries of a number of towns as to the system adopted in determining whether a free midwife should be granted.

The Town Clerk has now ascertained the following particulars:

*Nottingham.*—Grants for free midwives have been made in some very few cases, and have been determined by the question of poverty. No scale or table on which such grants are made has been adopted.

*Bradford.*—A free midwife is not granted in more than 1 per cent. of the cases attended. No scale or table is used in determining whether the charge shall be imposed or not, but each case is considered on its merits.

*Manchester* has the following scale of incomes upon which payments are made to medical practitioners called in on the advice of midwives under the Midwives Act, 1902:

| | Per week | | Per week |
|---|---|---|---|
| Man and wife ... | 35/- | With four children... | 43/- |
| With one child ... | 37/- | With five ,, ... | 45/- |
| With two children .. | 39/- | With six ,, ... | 47/- |
| With three ,, ... | 41/- | With seven ,, ... | 49/- |

*Leeds.*—No provision of free midwives has been adopted. The Health Committee has adopted a scale upon which to estimate the proportion of the fees of a medical man called in by midwives in emergency cases, which should be paid by the patients. This scale is:

| Average weekly income per head per week of family. | Proportion paid by Health Committee | Proportion paid by patient. |
|---|---|---|
| 5/- | Whole | Nothing |
| 6/- | 5/6ths | 1/6th |
| 7/- | 5/7ths | 2/7ths |
| 8/- | 5/8ths | 3/8ths |
| 9/- | 5/9ths | 4/9ths |
| 10/- | Half | Half |
| 12/- | 5/12ths | 7/12ths |
| 14/- | 5/14ths | 9/14ths |
| 16/- | 5/16ths | 11/16ths |
| 18/- | 5/18ths | 13/18ths |
| 20/- | Nothing | Whole |

The rent is deducted from gross earnings before the average is calculated.

*Stoke-on-Trent* pays a fee of 5s. to the midwife in all cases in which she has been unable to obtain payment for her services in families in which the income does not exceed 21s. a week, and consists of a man and wife, and when the income does not exceed 23s. per week of a family of a man, wife, and child. As a matter of fact, there have been very few applications for this payment, and in future, should any application be received from a midwife, each case, with full particulars, will be submitted to the Health Committee for consideration on its merits.

*Bristol* has not adopted a scheme for the provision of free midwifery treatment.

*Sheffield.*—The Corporation has never yet been asked to supply a free midwife.

*Liverpool.*—No scale. In all cases where a midwife has been called in and a patient is unable to pay, the Committee has agreed to pay a fee of 10s. on the certificate of the Medical Officer of Health.

*West Ham.*—No arrangements made for the granting of a free midwife in necessitous cases.

*Leicester.*—The Council pays the midwives' fees, or half-fees, in necessitous cases. Each case is decided on its merits. In practice, it is only usual to pay the whole fee if there is no maternity benefit coming in.

*Salford* Corporation does not grant free midwives in necessitous cases.

Arising out of the foregoing facts, the Hull Committee has arranged that free midwives be only granted after the cases have been thoroughly investigated, and only to those persons coming within the following scale of weekly income of family:

| | | | |
|---|---|---|---|
| Man and wife ... | 20/- | With five children... | 38/2 |
| With one child ... | 22/6 | With six ,, ... | 42/1 |
| With two children... | 26/5 | With seven ,, ... | 46/- |
| With three ,, ... | 30/4 | With eight ,, ... | 50/- |
| With four ,, ... | 34/3 | | |

These incomes are after excluding rent and insurance.

The question of granting a free midwife in cases where maternity benefits are due has been deferred.

